# Ketone Supplementation in Trained and Physically Active Individuals: Effects on Athletic Performance and Metabolic Variables—A Systematic Review

**DOI:** 10.3390/life16071147

**Published:** 2026-07-10

**Authors:** Jose Martí-Martí, Dídac Navarro-Martínez, Tamara Álvarez-Segura, Juan Bautista Miñana, Consuelo Moratal, Javier Zahonero

**Affiliations:** 1 Department of Physical Preparation and Conditioning, Faculty of Physical Activity and Sport Sciences, Catholic University of Valencia, 46900 Torrent, Spain; jose.marti@ucv.es (J.M.-M.); tamara.alvarez@mail.ucv.es (T.Á.-S.); jb.minana@ucv.es (J.B.M.); consuelo.moratal@ucv.es (C.M.); javier.zahonero@ucv.es (J.Z.); 2 Doctoral School, Catholic University of Valencia San Vicente Mártir, 46001 Valencia, Spain

**Keywords:** ketones, β-hydroxybutyrate, exogenous ketones, metabolism, endurance performance, nutritional ergogenic aids, systematic review

## Abstract

Exogenous ketone supplementation has emerged as a potential ergogenic strategy for trained and physically active individuals, prompting increasing research into its effects on exercise performance and metabolism. This systematic review evaluated the effects of ketone supplementation on athletic performance, metabolic variables, and physiological responses in trained and physically active populations. Searches were conducted in EBSCOhost, PubMed, and Web of Science following PRISMA 2020 guidelines. Peer-reviewed controlled studies published between 2018 and December 2025 were included. Twenty-six studies met the eligibility criteria. Ketone supplementation consistently increased circulating β-hydroxybutyrate concentrations and modified glucose, lactate, and acid–base balance. Effects on exercise performance were heterogeneous, with some studies reporting improvements in time-trial performance and cognitive function, whereas others found no benefit or even impaired performance. Co-ingestion with sodium bicarbonate attenuated metabolic acidosis and occasionally enhanced performance outcomes. Ketone supplementation also influenced cardiorespiratory responses and fluid regulation, and may promote muscular angiogenesis during periods of training overload. Overall, current evidence remains heterogeneous and does not support definitive conclusions regarding ergogenic efficacy, which appears to depend on exercise intensity, participant characteristics, supplementation protocols, and co-ingestion strategies.

## 1. Introduction

Endogenous ketone bodies, particularly β-hydroxybutyrate (β-HB), are produced in the liver during periods of energy stress, such as prolonged fasting, carbohydrate restriction, or sustained aerobic exercise, and serve as alternative oxidative substrates for peripheral tissues, including skeletal muscle and the brain [1,2]. Exogenous ketone supplementation, in the form of ketone esters (KE), ketone monoesters (KME), or ketone salts (KS), has emerged as a strategy to acutely elevate circulating β-HB concentrations without the need for prolonged dietary restriction [1,2]. The biological rationale for their use in endurance sports is grounded in their potential to alter substrate metabolism during prolonged exercise by reducing dependence on limited glycogen stores, increasing fat oxidation, and providing an additional oxidative fuel source for working muscles [2,3,4].

Beyond substrate metabolism, ketone supplementation has been hypothesized to influence endurance performance through several mechanisms, including improved exercise economy, modulation of central fatigue, altered cardiorespiratory and cardiac responses, and activation of cell-signalling pathways involved in training adaptation (e.g., mTORC1, AMPK) [2,3,5,6]. However, empirical evidence supporting these putative benefits remains inconsistent. A foundational study by Cox et al. (2016) [4] reported improvements in endurance performance during a 60-min steady-state cycling protocol at approximately 75% VO_2_max with elevated blood β-HB concentrations. In contrast, several subsequent randomized controlled trials (RCTs) have failed to replicate this finding, reporting no significant improvements in time-trial performance, time-to-exhaustion, or maximal oxygen uptake following acute or repeated ketone supplementation [7,8,9].

Multiple narrative and systematic reviews have attempted to synthesize this growing body of literature with mixed results. Pinckaers et al. (2017) [1] described ketone bodies as the “next magic bullet” while cautioning that the initial evidence was limited and that methodological heterogeneity precluded definitive conclusions. Valenzuela et al. (2021) [10] similarly concluded that exogenous ketone supplementation does not consistently improve endurance performance, attributing inconsistent findings to gastrointestinal distress, individual variability, and heterogeneity in study designs. Harvey et al. (2019) [11] and Ashtary-Larky et al. (2022) [12] focused on ketogenic diets rather than exogenous supplementation, emphasizing that endogenous nutritional ketosis may confer different physiological effects than acute exogenous ketone ingestion. More recently, Sun et al. (2025) [13] published a systematic review on ketogenic diets and ketone supplements in endurance runners, concluding that the current evidence does not support consistent ergogenic effects, with most primary studies showing no significant benefit and a few reporting performance impairment. Sitko (2024) [8] examined the role of exogenous ketones in road cycling and noted that the limited number of well-controlled studies, small sample sizes, and heterogeneity in supplementation protocols (type of ketone, dose, timing, co-ingestion with carbohydrate or bicarbonate) constrain the generalizability of the findings.

However, several key uncertainties persist in the literature. First, the heterogeneity in outcomes across studies—ranging from performance improvement to impairment—remains poorly understood, with no clear consensus on the conditions (exercise modality, dose, and population) under which benefits may emerge [5,7,8,9]. Second, most studies have focused on male endurance athletes, predominantly trained cyclists, limiting the generalizability of the findings to female athletes, team-sport athletes, occupational groups with high physical demands (e.g., firefighters), and recreationally active individuals [8,14,15]. Third, the effects of repeated or chronic ketone supplementation on training adaptation (e.g., muscle capillarization, mitochondrial biogenesis, and recovery) have only begun to be explored, with few available studies reporting context-specific findings [5,6,16]. Fourth, the safety profile of chronic exogenous ketone use, including potential gastrointestinal distress and effects on acid–base balance, remains insufficiently characterized [8,10]. Finally, prior reviews have generally provided narrative syntheses but have not consistently applied formal tools to grade the certainty of evidence (e.g., GRADE) or to integrate the appraisal of methodological quality (e.g., JBI) with effect direction conclusions [1,10,13].

To address these gaps, the present systematic review aimed to: (1) comprehensively synthesize the available evidence on the effects of exogenous ketone supplementation on athletic performance, metabolic variables, and physiological responses in trained and physically active individuals; (2) extend the scope of prior reviews by including studies published up to December 2025, incorporating four population subgroups (trained endurance athletes, team-sport athletes, occupational groups, and recreationally active individuals) as defined by the PICOS strategy, and distinguishing acute from repeated/chronic supplementation protocols; (3) evaluate the methodological quality of included studies using the Joanna Briggs Institute (JBI) critical appraisal tools for both randomized and non-randomized/quasi-experimental designs; and (4) assess the certainty of evidence using a GRADE-informed narrative approach, adapted for systematic reviews without meta-analysis, to provide transparent and reproducible ratings across multiple outcomes, including endurance performance, β-HB concentrations, lactate, respiratory exchange ratio, and heart rate.

Given the growing relevance of this topic in the sports domain and the need to provide solid scientific evidence to guide supplementation practices in endurance athletes, the importance of conducting a comprehensive systematic review is evident. This systematic review aimed to evaluate the effects of exogenous ketone supplementation on endurance performance and metabolic responses in trained and physically active populations, including endurance and team-sport athletes, occupational groups with high physical demands (e.g., professional firefighters), and recreationally active individuals, all of whom are considered to share a baseline of structured training or regular physical activity.

To achieve this, a comprehensive search of scientific databases was conducted to identify relevant studies on ketone supplementation in endurance athletes. The review analyzed supplementation protocols, their effects on sports performance, and the methodological quality of the included studies.

## 2. Materials and Methods

### 2.1. Eligibility Criteria

The research question guiding this systematic review was: “Does ketone supplementation provide performance benefits for individuals engaged in endurance training or physically active populations?”. This systematic review was conducted and reported in accordance with the PRISMA 2020 (Preferred Reporting Items for Systematic Reviews and Meta-Analyses) guidelines [17] and a completed PRISMA checklist and detailed search strategy have been provided (Supplementary Table S1 and Appendix A). The search was conducted up to 1 December 2025. This systematic review was not prospectively registered in PROSPERO; however, the protocol and methodological framework were documented in advance and are publicly available via the Open Science Framework (OSF), with efforts made to ensure transparency and minimize reporting bias (https://osf.io/sjezx) (accessed on 1 March 2026) before the initiation of the literature search. This limitation is acknowledged, as the absence of prospective registration may increase the risk of selective reporting or post hoc methodological modifications. However, the review process followed a predefined analytical framework to minimize potential bias in the selection, extraction, and synthesis of evidence. This research question was formulated according to the PICOS strategy: Population (P): The target population comprised endurance athletes, defined as individuals engaged in structured training aimed at optimizing prolonged aerobic performance, typically characterized by elevated cardiorespiratory fitness (e.g., VO_2_max ≥ 50 mL/kg/min) and regular participation in endurance sports, such as cycling, long-distance running, or triathlon. Additionally, the review included other physically active populations to enhance ecological validity: (1) team-sport athletes (e.g., soccer and rugby players) engaged in intermittent high-intensity exercise; (2) occupational groups with physically demanding job requirements (e.g., professional firefighters); and (3) recreationally active individuals defined as those participating in regular physical activity or meeting a minimum cardiorespiratory fitness threshold (e.g., VO_2_max or VO_2_peak ≥ 50 mL·kg^−1^·min^−1^) but not meeting the criteria for trained athletes. All participants were required to demonstrate adequate cardiovascular fitness through objective measures, such as VO_2_max, training history, or competitive experience. Intervention (I): The intervention consisted of either acute (single-dose) or repeated/chronic administration of exogenous ketone supplements—ketone bodies or precursors given orally to raise blood ketone levels independently of fasting or carbohydrate restriction; these intervention types were treated as distinct categories and were not interpreted as a single pooled effect. Comparison (C): Comparison conditions included placebos matched for taste, color, texture, and energy content; carbohydrate-based controls (e.g., isoenergetic sports drinks); water-only controls; or no-intervention groups when appropriate. Studies lacking a comparison group were excluded, except in justified within-subject or baseline animal designs. In crossover studies, the comparison condition served as the within-subject control, with a minimum 48-h washout period between interventions for acute supplementation trials. Outcome (O): Primary outcomes were those focused on endurance performance measures such as time to exhaustion or time-trial completion time. Secondary outcomes included physiological variables influencing performance, cardiovascular responses, metabolic markers (lactate, β-hydroxybutyrate) or cognitive performance during exercise. Endurance performance outcomes were derived from both maximal and submaximal exercise protocols; these were considered as distinct categories and were not interpreted as a single unified performance outcome.

The inclusion criteria were as follows: original research articles published in peer-reviewed scientific journals from 2018 were considered for inclusion. Studies published before 2018 were excluded unless they provided foundational mechanistic insights that were not adequately addressed in more recent publications. No language restrictions were applied. Human intervention studies (randomized and nonrandomized) that met the intervention criteria were included.

The exclusion criteria were: Studies examining ketone metabolism in clinical populations (e.g., patients with metabolic syndrome, ketotic conditions, or chronic diseases affecting exercise or ketone metabolism; also patients with neuromuscular diseases) were excluded to ensure consistency with the focus on endurance athlete populations. Finally, studies that were systematic reviews or meta-analyses and doctoral theses were excluded.

### 2.2. Information Sources

The literature search was conducted across different electronic databases to ensure comprehensive coverage of the relevant scientific literature. The primary database searched were PubMed and EBSCOhost, which provides access to multiple databases including SPORTDiscus, CINAHL, and MEDLINE. An additional search in Web of Science was conducted. An structured and methodologically rigorous search strategy was applied to identify relevant studies. The search was conducted without language restrictions; however, a publication date limit from 2018 onward was applied in accordance with the predefined eligibility criteria. Additionally, the reference lists of studies selected during the initial screening phase were carefully examined to identify any further relevant publications that may not have been captured in the primary search.

### 2.3. Search Methods for Identification of Studies

The search terms were developed using a combination of medical subject headings (MeSH terms) and free-text keywords to maximize search sensitivity while maintaining specificity. The search strategy incorporated terms related to exogenous ketone administration and terms related to exercise and sports performance. The Boolean operators AND and OR were used to combine search terms effectively. The following search terms were applied (((“exogeneous” OR “exogeneously” OR “exogenic” OR “exogenous” OR “exogenously”) AND (“ketosis” OR “ketosis”)) OR (“keton” OR “ketones” OR “ketones” OR “ketone” OR “ketones” OR “ketonic” OR “ketonization” OR “ketonized”)) AND (“sport s” OR “sports” OR “sports” OR “sport” OR “sporting”). The complete search strategy is detailed in Appendix A, where the PUBMED database is presented as an example, to ensure transparency and reproducibility. The records were screened for eligibility by two independent reviewers, and any disagreements were resolved by consensus. All retrieved records were screened according to predefined eligibility criteria.

The selection of search terms was informed by an analysis of how the relevant literature is indexed in PubMed. In MeSH, β-hydroxybutyrate and acetoacetate are indexed under the heading ‘3-Hydroxybutyric Acid’ (D020155) and the broader tree ‘Ketone Bodies’ (D007657), both of which are automatically retrieved by the free-text term ‘ketones’ through PubMed’s automatic term mapping. Likewise, concepts related to endurance and athletic performance are indexed under the MeSH heading ‘Sports’ (D013177) and its subheadings (including ‘Physical Endurance’ and ‘Sports Performance’), which are retrieved by the free-text term ‘sport’. The phrase ‘exogenous ketosis’ is rarely used as a primary indexing term, with authors more commonly describing their interventions as ‘exogenous ketone supplementation’ or ‘ketone ester/salt ingestion’, wording already captured by the ‘exogenous’ and ‘ketones’ components of the original strategy. For these reasons, the original three-concept structure (exogenous/ketones/sport) was considered methodologically sufficient to retrieve the relevant body of evidence.

### 2.4. Study Selection Process

The study selection process followed a systematic two-phase screening approach to minimize selection bias and ensure the consistent application of eligibility criteria. In the first phase, two independent reviewers screened the titles and abstracts of all identified records according to the predefined eligibility criteria. Records were classified as (1) potentially eligible and requiring full-text screening, (2) ineligible based on title and abstract, or (3) uncertain and requiring clarification. Disagreements between reviewers at this phase were resolved through discussion, and records classified as uncertain were subjected to full-text screening.

In the second phase, the full texts of potentially eligible records were obtained and assessed for eligibility by the same two independent reviewers.

### 2.5. Data Extraction and Data Items

Two authors independently reviewed the data extracted from each study. If consensus was not reached, a third author was consulted to complete the data extraction form. The form was designed to capture all relevant information from each included study while ensuring consistency in the data collected: study identification, study design and methods, participant characteristics, intervention details, control/comparator details, exercise protocol, outcome measures and results.

#### Data Synthesis and Approach to Heterogeneity

Given the anticipated heterogeneity in exercise protocols, supplementation strategies, and participant characteristics, a structured narrative synthesis approach was used to organize and report the evidence, consistent with the current methodological guidance for systematic reviews without meta-analysis. The findings were tabulated and synthesized according to four pre-specified subgroup dimensions: (i) population subgroup, as defined in the PICOS strategy (trained endurance athletes, team-sport athletes, occupational groups, and recreationally active individuals); (ii) supplementation protocol (acute single-dose vs. repeated/chronic administration), which was treated as distinct categories and not pooled; (iii) ketone formulation (ketone monoester, ketone ester, or ketone salts, with or without co-ingestion strategies such as carbohydrate, sodium bicarbonate, caffeine, or taurine); and (iv) outcome type (endurance performance, metabolic variables including β-hydroxybutyrate, glucose, lactate, and acid–base balance, physiological responses including heart rate and respiratory exchange ratio, and cognitive function). This pre-specified subgroup structure allowed for a systematic examination of variability across studies and contexts.

Given the absence of pooled effect estimates in this narrative synthesis, a formal quantitative sensitivity analysis restricted to studies with low risk of bias was not performed, as there is no pooled statistic to re-estimate under restricted inclusion conditions. The methodological equivalent of this operation is integrated into the GRADE-informed certainty ratings (Section 2.6), where the ‘study limitations’ domain explicitly incorporates risk of bias across the body of evidence. Furthermore, the main findings were tabulated and discussed by individual study quality classification, enabling narrative inspection of whether conclusions remained consistent across studies with higher versus lower methodological rigor. This approach allows the impact of risk of bias on the certainty of evidence to be evaluated without requiring a quantitative pooled analysis.

### 2.6. Quality and Risk of Bias Assessment in Included Studies

Two authors independently appraised methodological quality.

The methodological quality of the included randomized controlled trials (RCTs) was critically appraised using the Joanna Briggs Institute (JBI) Critical Appraisal Checklist for Randomized Controlled Trials. This validated instrument comprises 13 domains that evaluate key methodological dimensions of RCTs, including randomization methods, allocation concealment, blinding procedures, treatment group comparability, outcome measurement integrity, follow-up completeness, intention-to-treat analysis, statistical appropriateness, and trial design validity [18]. Each included study was independently assessed by two reviewers, and discrepancies were resolved through discussion or consultation with a third reviewer. For each domain, studies were classified as “Yes,” “No,” “Unclear,” or “Not/Applicable” based on the adequacy of reporting and methodological rigor demonstrated in the published manuscript. Studies were not excluded based on methodological quality scores; rather, the appraisal findings were used to contextualize the synthesis of results and to assess the overall confidence in the evidence base.

The methodological quality of the included non-randomized and quasi-experimental studies was independently assessed by two reviewers using the Joanna Briggs Institute (JBI) Critical Appraisal Checklist for Quasi-Experimental Studies [19]. This tool encompasses nine domains, including the clarity of causal relationships, participant similarity, presence of a control group, and reliability of outcome measurements. Any discrepancies between the reviewers were resolved through consensus or consultation with a third reviewer. Studies were categorized based on their risk of bias, ensuring that only research meeting high methodological standards contributed to the final synthesis of evidence.

The certainty of evidence was assessed using a GRADE-informed narrative approach adapted for systematic reviews without meta-analysis [20]. The following domains were evaluated: study limitations (risk of bias), inconsistency, indirectness, imprecision, and publication bias. Each outcome was rated as ‘high,’ ‘moderate,’ ‘low,’ or ‘very low’ certainty, starting from ‘low’ given the absence of pooled effect estimates.

## 3. Results

### 3.1. Studies Selection

**Figure 1:**
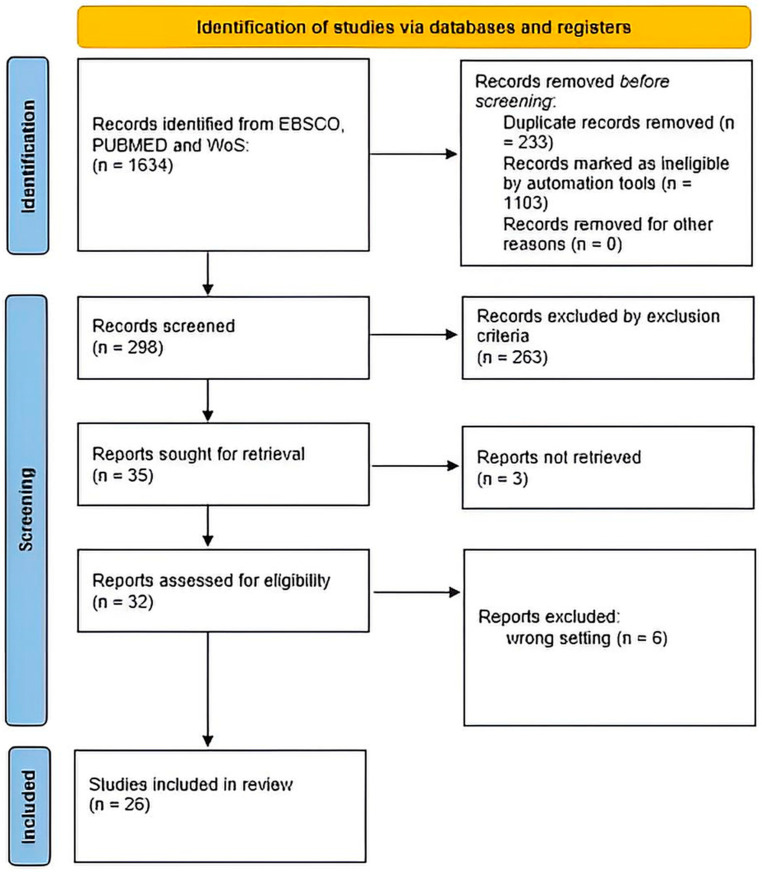
PRISMA flow diagram.

The initial search yielded 1634 articles. After removing duplicates and applying predefined inclusion criteria (articles published from 2018 to 1 December 2025, in humans), 1336 studies were excluded, resulting in 298 articles. After reviewing the articles, 263 studies were rejected based on the exclusion criteria. Finally, 26 studies fulfilled the inclusion criteria and were included in the systematic review. The study selection process is summarized in the PRISMA flow diagram (Figure 1). The included studies comprised both acute and repeated or chronic supplementation protocols, which were interpreted separately to avoid conflating immediate physiological responses with longer-term adaptations [21,22,23,24,25,26,27,28,29,30,31,32,33,34,35,36,37,38,39,40,41,42,43,44,45,46].

### 3.2. Characteristics of Included Studies

A total of 26 studies involving 380 participants were included in this review, predominantly utilizing experimental designs to evaluate the effects of exogenous ketosis on endurance performance, metabolic, physiological and cognitive variables. Research was conducted across several geographic regions, with the highest number of studies in Belgium (*n* = 7), the United States (*n* = 7), and the United Kingdom (*n* = 2). Other participating countries included Ireland, Canada, and France, and one multicenter study involved both the United States and Ireland.

The participant cohorts were largely composed of athletic populations, including trained cyclists, triathletes, professional rugby players and endurance runners, among others. While most studies focused exclusively on male participants, others included female cohorts or mixed sex groups. Notably, two studies focused specifically on women’s health. The sample sizes were relatively small, ranging from 6 to 32 participants per study, with a mean age generally falling between 18 and 35 years.

The interventions primarily involved the administration of ketone monoesters (KME) or ketone salts (KS), often compared with carbohydrate (CHO) placebos or calorie-free controls, such as water. Training protocols were diverse, ranging from acute high-intensity cycling time trials and simulated matches to 3-week endurance overload programs and 100-km trail runs. Regarding ethical transparency, nine studies explicitly reported a conflict of interest, ten studies declared no conflicts of interest, and eight studies did not provide this information. All the characteristics are included in Table 1.

### 3.3. Quality Assessment

The critical appraisal revealed a generally high overall methodological rigor across the included studies. All trials successfully implemented appropriate randomization and ensured baseline comparability between the intervention and control groups (Table 2). Blinding procedures for participants and personnel were consistently reported; however, a minor proportion of trials did not achieve full blinding of those delivering the treatment due to the distinct organoleptic properties of ketone supplements [23,24], which represents a potential source of performance bias that may have influenced the expectancy effects. Most studies appropriately utilized crossover designs with adequate washout periods and employed robust statistical methods to account for intra-individual variability. Regarding funding and sponsorship, nine studies explicitly reported a conflict of interest statement, ten declared no conflicts, and eight did not provide this information, precluding a definitive assessment of the industry’s influence on outcomes. The overall risk of bias was assessed as low to moderate, with methodological quality providing a reliable foundation for synthesizing the available evidence. However, the findings should be interpreted within the context of these acknowledged limitations.

Table 3 presents the risk of bias assessment for non-randomized studies investigating the effects of ketone supplementation on endurance athletic performance, utilizing the JBI Checklist for Quasi-Experimental Studies. The critical appraisal demonstrated a high level of methodological quality across quasi-experimental research. Both Evans et al. [25] and Clark et al. [22] achieved high-quality assessments with a JBI rating (≥7/8), indicating a low risk of bias. In both instances, the causal relationship between exogenous ketone salt ingestion and metabolic or performance outcomes was explicitly stated. Furthermore, these studies utilized robust crossover designs that ensured participant similarity and consistent measurement protocols across experimental conditions. The reliability of the evidence was further supported by the use of validated physiological measures, such as the respiratory exchange ratio (RER) and blood metabolites, alongside appropriate statistical modeling to account for repeated measures. Across the checklist, we found that the weaknesses concerned the existence of an external control group (crossover design relied on within-subject comparisons without a parallel group receiving no intervention).

### 3.4. Certainty of Evidence

The certainty of the evidence varied across the outcomes (Supplementary Table S2). The effect of ketone supplementation on β-hydroxybutyrate concentrations was supported by moderate-certainty evidence, whereas performance outcomes (time-trial, time to exhaustion), heart rate, and cognitive function were supported by low-certainty evidence due to substantial heterogeneity, small sample sizes, and potential publication bias.

### 3.5. Outcomes by Type of Study

#### 3.5.1. Synthesis of Findings by Population, Intervention, and Outcome

To facilitate a coherent and thematically organized synthesis, the presentation of the findings in this section is structured according to three pre-specified dimensions consistent with the PICOS strategy: (i) supplementation timing (acute single-dose vs. repeated/chronic administration), (ii) exercise modality (sustained endurance exercise vs. intermittent high-intensity exercise), and (iii) primary outcome type (endurance performance, metabolic variables, physiological responses, and cognitive function). This structure was applied consistently throughout the analysis to enable a direct comparison of the effects across contexts. The only finding consistent across all RCTs, regardless of these dimensions, is that ketone supplementation (particularly with ketone monoesters and ketone esters) induces a rapid and marked increase in circulating β-HB concentrations.

When organized according to the three pre-specified dimensions, three consistent patterns emerged across the included evidence: (i) acute single-dose supplementation consistently elevated β-hydroxybutyrate and modified substrate oxidation across all exercise modalities, but yielded inconsistent effects on endurance performance—with no benefit or impairment in most trained endurance athletes, occasional benefits in team-sport simulations, and reduced heart rate in occupational submaximal exercise; (ii) repeated or chronic supplementation (≥7 days) demonstrated more consistent effects on training adaptation outcomes, including enhanced muscle capillarization and improved recovery markers during endurance overload, while performance effects remained variable; and (iii) exercise modality moderated the response, with sustained high-intensity endurance protocols showing predominantly null or negative performance effects, while intermittent and occupational protocols occasionally showed benefits. These patterns support the conclusion that ketone supplementation produces robust metabolic effects, the translation of which into ergogenic benefits is highly context-dependent.

Methodological context. These findings should be interpreted in light of the JBI critical appraisal (Section 3.3), which indicated generally high methodological quality across the included RCTs, with consistent implementation of randomization and baseline comparability, but with some limitations in blinding of participants and personnel due to the organoleptic properties of the ketone supplements, as well as incomplete reporting of conflict-of-interest statements in a subset of studies. These quality considerations are reflected in the GRADE certainty-of-evidence ratings (Section 3.4), which downgrade performance and physiological outcomes, primarily for inconsistency and imprecision.

Detailed individual study findings (presented in full in Table 4) include specific context-dependent effects: reduced heart rate during submaximal occupational exercise in firefighters [29]; improved high-intensity cycling performance with ketone ester + sodium bicarbonate co-ingestion [43]; small cognitive benefits (reaction time, vigilance) during intermittent exercise and team-sport simulations [33,46]; maintained alertness and reduced inflammatory markers during a 100-km trail race [38]; and enhanced muscle capillarization following 3-week ketone ester supplementation during endurance training overload [38]. These detailed findings are consistent with the three patterns identified in the thematic synthesis above and support the context-dependent nature of the ergogenic effects of ketone supplementation.

#### 3.5.2. Complementary Evidence from Non-Randomized and Single-Blind Studies

Consistent with RCT findings, non-randomized evidence indicates that acute ketone salt ingestion produces only modest β-hydroxybutyrate elevations and does not translate into measurable performance benefits in trained endurance athletes. The JBI appraisal of these two non-randomized studies (Table 3) classified both as having high methodological quality, although the absence of random allocation is an inherent design limitation that restricts causal inference. Accordingly, non-randomized evidence should be considered complementary and hypothesis-generating rather than confirmatory, reinforcing the conclusions drawn from the larger RCT body that ketone supplementation—particularly with ketone salts—does not consistently improve endurance performance.

Detailed findings from non-randomized studies (Table 4) showed that acute ketone salt ingestion (0.3–0.38 g·kg^−1^) in trained cyclists produced modest β-hydroxybutyrate elevations, reduced blood glucose, and increased respiratory exchange ratio, but did not improve time-trial, steady-state, or high-intensity cycling performance [22,25]. Co-strategies such as whole-body cooling also failed to enhance performance.

## 4. Discussion

This systematic review included 26 studies that investigated the effects of exogenous ketone supplementation on endurance performance and metabolic variables. The main findings can be summarized as follows: ketone supplementation consistently elevated circulating β-hydroxybutyrate concentrations and altered metabolic variables, but performance outcomes were heterogeneous, with the majority of randomized controlled trials reporting no significant improvements in time-trial performance, time to exhaustion, or maximal workload; contextual benefits were observed under specific conditions, such as intermittent high-intensity exercise, ultra-endurance events, and training overload periods; ketone co-administration with sodium bicarbonate attenuated acidosis and occasionally enhanced outcomes; and acute ketone salt ingestion produced limited ergogenic benefits compared to ester formulations. These findings are discussed in detail below under distinct subheadings addressing the differential effects of acute versus chronic supplementation and comparisons across different physically active populations. A major source of heterogeneity across studies is the inclusion of both acute and repeated or chronic supplementation designs, which represent fundamentally different physiological contexts and should not be interpreted as being equivalent. Part of this variability may be explained by the inclusion of both maximal and submaximal exercise protocols, which represent different physiological demands and should not be interpreted as equivalent.

These studies encompassed small-to-moderate samples (6–32 participants), predominantly comprising young adults (18–35 years) with varied training backgrounds. Some protocols even included dietary controls, such as ensuring protein intake below 0.8 g·kg^−1^·day^−1^. This wide variability highlights the growing interest in ketone supplementation across different athletic populations and justifies the need for a comprehensive systematic review of the literature.

The effects of exogenous ketone supplementation on endurance performance were inconsistent across the included studies. Most randomized controlled trials reported no significant improvements in time-trial performance, time to exhaustion, or maximal workload. In some cases, performance impairments were observed, particularly during sustained, high-intensity exercise. Only a limited number of studies have demonstrated improvements, typically under specific conditions, such as intermittent high-intensity exercise or combined supplementation strategies. These findings indicate that despite occasional positive results, there is no consistent evidence supporting a clear ergogenic effect of ketone supplementation on endurance performance.

In contrast to performance outcomes, ketone supplementation consistently increased circulating β-hydroxybutyrate concentrations and altered several metabolic variables, including reduced blood glucose levels and modifications in acid–base balance. Changes in lactate levels, respiratory exchange ratio, and cardiovascular responses have also been frequently reported. However, these metabolic adaptations should be interpreted as physiological responses, rather than direct indicators of improved performance. Evidence shows that such changes do not consistently translate into enhanced endurance outcomes and should therefore be considered secondary and mechanistic in nature.

### 4.1. Supplementation Protocols and Administration Strategies

The studies included in this review demonstrated considerable diversity in their supplementation protocols. The most commonly used forms were ketone salts and ketone esters, with ketone monoesters, particularly (R)-3-hydroxybutyl (R)-3-hydroxybutyrate, being the most prevalent. For ketone salts, the typical dose was approximately 7 g, whereas for ketone esters, the formulations varied widely depending on the study objectives. Evidence from Dearlove et al. [23] indicates that doses of approximately 252 mg·kg^−1^ induce mild ketosis, whereas doses near 752 mg·kg^−1^ produce higher βHB concentrations.

There was no consensus across studies regarding timing or dosing strategies. Some trials administered ketones as a single pre-exercise dose, while others used multiple doses before, during, and after exercise or even before sleep. All randomized trials included visually and sensorially matched placebo conditions to maintain the blinding.

Several studies have investigated co-ingestion strategies, such as ketone esters combined with sodium bicarbonate, caffeine, or beta-alanine [37,47]. These combinations aim to enhance buffering capacity or synergize with known ergogenic aids. The typical dosages ranged from 50–75 g of ketone ester and 180–300 mg·kg^−1^ of sodium bicarbonate.

The exercise protocols also varied substantially among the studies. Cycling studies frequently employ incremental exercise tests until exhaustion, long-duration intermittent efforts followed by time trials, or combined endurance-sprint tests, including Wingate assessment [40]. Running studies used treadmill efforts at fixed percentages of VO_2_max, followed by 10-km time trials. In soccer [41], a simulation was developed to replicate a 45-min match comprising intermittent, sport-specific activity blocks.

### 4.2. Effects of Acute Supplementation and Repeated or Chronic Supplementation

#### 4.2.1. Acute Supplementation

The most robust evidence in this review stems from randomized, double-blind, controlled trials investigating acute ketone supplementation [21,22,23,24,25,26,30,31,32,33,34,35,36,37,40,41,42,45,46]. These studies consistently demonstrated that a single dose of ketone monoester or ketone salt rapidly elevates circulating β-hydroxybutyrate concentrations, often exceeding 1–3 mM within 30–90 min of ingestion. Simultaneously, ketone supplementation reduces blood glucose and lactate levels, modifies the acid–base balance through metabolic acidosis, and alters substrate utilization patterns. However, these metabolic changes do not uniformly translate to performance improvements.

The cardiorespiratory and physiological responses were heterogeneous. McCarthy et al. (2021) [30] reported increased cardiorespiratory stress markers at submaximal exercise intensities, while McCarthy et al. (2023) [32] observed a modest increase in heart rate without changes in cardiac output, accompanied by a reduction in maximal workload. These findings are consistent with previous evidence showing that ketogenic diets can affect heart rate variability in obese individuals [48] and that ketone administration influences cardiovascular variables, including heart rate and blood pressure, in healthy participants [49]. Another recurring finding was the alteration in the acid–base balance. Poffé et al. (2021) [35,36] and Ramos-Campo et al. (2024) [42] demonstrated that ketone ester supplementation can induce metabolic acidosis, characterized by reductions in blood pH and bicarbonate concentrations, potentially impairing performance unless counteracted by sodium bicarbonate co-ingestion. These effects are thought to result from the suppression of glycolytic flux and the consequent acid–base disturbances induced by ketone esters, whereas sodium bicarbonate restores acid–base homeostasis and may facilitate ergogenic benefits [4]. Each mole of βHB oxidized generates approximately one mole of H^+^, contributing to the measurable decrease in blood pH (~0.10 units) reported with ketone ester ingestion. Sodium bicarbonate, by buffering these protons, restores acid–base homeostasis and unmasks the ergogenic potential of ketone oxidation, as demonstrated by Poffé et al. [35,36] and Ramos-Campo et al. [42]. This interaction exemplifies how a co-ingestion strategy can shift the dose–response relationship of ketone supplementation by removing a key mechanistic constraint. Practically, this implies that bicarbonate co-ingestion should be considered a default component of any high-intensity ketone supplementation protocol rather than an optional add-on. Furthermore, under hypoxic conditions, Poffé et al. (2021) [37] showed that ketone esters attenuated arterial oxygen desaturation and improved muscle oxygenation in healthy individuals. Similar adaptations have been observed in animal models of mitochondrial dysfunction and oxidative stress, where ketogenic diets increases mitochondrial biogenesis and oxygen consumption rates [50].

The apparent intensity-dependent modulation of ketone efficacy may be explained by converging physiological constraints. At high exercise intensities (>75% VO_2_max), the rate of ATP demand exceeds the capacity of oxidative phosphorylation alone, requiring substantial contribution from anaerobic glycolysis. Under these conditions, the metabolic acidosis induced by ketone ester metabolism (βHB^−^ → AcAc^−^ + H^+^) becomes particularly detrimental, as it inhibits key glycolytic enzymes (phosphofructokinase) and impairs calcium handling by the sarcoplasmic reticulum, thereby reducing force production. In contrast, during submaximal or intermittent efforts, the proportionally lower glycolytic flux permits ketone co-oxidation without severe acidosis, allowing the energetic and oxygen-sparing benefits of βHB oxidation to manifest more fully. This biochemical framework predicts a narrow and context-specific ‘ergogenic window’ for ketone supplementation, consistent with the heterogeneous results observed across the included studies.

Significant improvements have been observed in endurance and performance using specific protocols. For example, Poffé et al. (2020) [34] demonstrated that the use of a KE drastically alters glucose and lactate metabolism, increasing the mean power during time trials (TT15′) and prolonging the time to exhaustion in final sprints. Along these lines, Quinones and Lemon (2022) [40] reported benefits in 20 km time trial performance and peak power. However, this efficacy is not universal, as studies by Evans et al. (2019) [26] and Waldman et al. (2024) [46] found no significant improvements in 10 km trials, while McCarthy et al. (2023) [31] identified a reduction in maximal workload and time trial performance following monoester ingestion; and when analyzing the effect in different disciplines, no effect has been found either [9].

From a metabolic and efficiency perspective, evidence suggests that ketones can influence exercise economy in a conditional manner. Dearlove et al. (2021) [24] indicated that BHB oxidation during exercise is influenced by the availability of other substrates, observing that running economy improves when KE is administered without carbohydrates, although this advantage disappears under co-ingestion conditions.

Some studies have demonstrated improvements in vigilance, processing speed, and reaction time following ketone ingestion, especially during fatiguing or intermittent exercise. For example, Quinones and Lemon (2022) [41] reported that ketone monoester ingestion improved cognitive performance during a simulated soccer match after inducing mental fatigue. Waldman et al. (2024) [46] observed that ketone monoesters with carbohydrates improved cognitive measures post-exercise in trained females. These findings suggest a potential role for dual-task or decision-based sports. Despite this, some authors have reported no cognitive effects during high-intensity exercise [45], reinforcing that context remains crucial, as the ketogenic diet seems to have a positive effect on executive functions, such as working memory, reference memory, and attention [51].

The two non-randomized or single-blind studies included in this review recruited trained cyclists and explored the effects of acute ketone salt ingestion at doses between 0.3–0.38 g·kg^−1^. Clark et al. (2021) [22] and Evans et al. (2018) [25] reported that plasma βHB increased modestly, blood glucose decreased, and both respiratory exchange ratio and heart rate were elevated during low-to-moderate exercise intensities. These studies also suggest a dose–response relationship between βHB concentrations and metabolic shifts. However, neither study reported improvements in endurance performance or time trial outcomes. Even when whole-body cooling was incorporated as an additional variable [22], no enhancement in power output or performance was observed in the latter. Taken together, these findings reinforce the conclusion that ketone salts are unlikely to provide meaningful ergogenic benefits during endurance exercises.

Team-sport athletes engaged in intermittent high-intensity exercise demonstrated more consistent benefits. Peacock et al. (2022) [33] observed that ketone monoester ingestion altered metabolism and simulated rugby performance in professional athletes. Specifically, ketone supplementation improved metabolic responses during rugby simulation, although the effects on physical performance were variable.

Although randomized controlled trials consistently demonstrate that ketone supplementation produces robust metabolic shifts (elevating βHB levels, reducing glycolysis, and altering acid–base balance), these physiological changes do not translate into measurable performance benefits in trained athletes. This dissociation between mechanistic and ergogenic outcomes highlights the importance of context-specific factors in sports nutrition [4,9].

#### 4.2.2. Repeated or Chronic Supplementation

In contrast, repeated or chronic supplementation protocols provide insights into long-term effects beyond acute metabolic changes. McAllister et al. (2019) [29] conducted a 7-day supplementation protocol where firefighters consumed ketone salts twice daily and documented a reduction in heart rate during submaximal occupational exercise wearing personal protective equipment, suggesting potential applications in physically demanding occupational contexts.

Regarding recovery and adaptation, Jameson et al. (2022) [28] reported a decrease in the levels of the cytokine TRAIL following repeated ketone supplementation after muscle-damaging eccentric exercises. Robberechts et al. (2022) [44] observed a reduction in NT-proANP secretion and suppression of diuresis during exercise, suggesting a direct impact on fluid regulation with repeated supplementation.

In contrast to the limited acute performance effects, sustained or intermittent supplementation appears to favor chronic adaptation. Poffé et al. (2023) [38] highlighted that intermittent exogenous ketosis during periods of training overload—achieved through postexercise and presleep ketone ester supplementation over a 3-week period—can stimulate angiogenesis in skeletal muscles by increasing proangiogenic factors, suggesting a potential benefit for long-term endurance adaptations. Poffé et al. (2023) [39] also reported increased plasma dopamine concentration and maintained mental alertness during a 100-km trail run when ketones were administered before, during, and after an ultra-endurance event, suggesting advantages under conditions where fatigue and neurocognitive decline are prominent. Jameson et al. (2022) [28] reported a decrease in the levels of the cytokine TRAIL following repeated ketone supplementation after muscle-damaging eccentric exercise, indicating anti-inflammatory effects that may facilitate muscle recovery. Robberechts et al. (2022) [44] observed a reduction in NT-proANP secretion and suppression of diuresis during exercise, suggesting direct effects on fluid regulation that may benefit athletes training in variable environmental conditions. These multilevel adaptations, ranging from molecular signaling to tissue-level angiogenesis, provide a mechanistic basis for the enhancement of training adaptations by chronic supplementation, even when acute performance benefits are not observed.

Robberechts et al. (2023) [43] observed that exogenous ketosis improved sleep efficiency and counteracted the decline in REM sleep after strenuous exercise when ketones were consumed before sleep and during surrounding training sessions, indicating potential recovery benefits with repeated supplementation.

Hiroux et al. (2023) [27] investigated the effects of a 4-week supplementation period during caloric restriction but focused on body composition and resting energy expenditure rather than acute performance outcomes.

At the cellular level, ketone esters lower blood pH by ~0.10 units through βHB^−^ metabolism, causing K^+^ influx and Na^+^ efflux [2], while simultaneously activating mTORC1 signaling, suppressing AMPK phosphorylation, and reducing pro-inflammatory cytokines, thereby establishing a molecular environment that favors post-exercise recovery and skeletal muscle remodeling through increased protein synthesis [52]. Additional findings include evidence of enhanced muscular angiogenesis during periods of endurance training overload, increased plasma dopamine during ultra-endurance running, and suppression of AMPK phosphorylation for several hours after exercise [53].

#### 4.2.3. Synthesis and Comparison with Existing Literature

Collectively, these results suggest that ketones may exert significant metabolic and signaling effects, even when their direct ergogenic influence is limited. While acute supplementation generally provides limited or inconsistent performance benefits, with some studies even reporting impairments, chronic or repeated supplementation appears to promote physiological adaptations, including enhanced muscle angiogenesis, improved exercise capacity under metabolic stress, and potential cognitive and recovery benefits. The physiological response to ketones includes alterations in internal homeostasis and recovery processes that could favor a faster restoration of psychocognitive and physical functions after strenuous exercise. The variability in results could be due to the fact that physiological changes during training also depend on diet, individual body composition, and metabolism [54], making it necessary to consider these variables when analyzing data. Our findings align with systematic reviews by Margolis and O’Fallon (2020) [55], and the meta-analysis by Valenzuela et al. (2020) and Brooks et al. (2022) [9,56], all concluding null or limited effects of ketone supplementation on endurance performance. The contrasting effects of acute versus chronic supplementation likely reflect fundamentally different biological processes. Acute responses are dominated by immediate substrate-level effects: increased circulating βHB availability, transient shifts in substrate oxidation, and acute acid–base disturbances. In contrast, repeated or chronic exposure activates slower genomic and cellular signaling pathways, including mTORC1 activation, AMPK suppression, and increased expression of pro-angiogenic factors such as VEGF [38]. The 3-week endurance training overload protocol by Poffé et al. [38] demonstrated that intermittent exogenous ketosis can potentiate training-induced capillarization, suggesting that ketones may act as a metabolic ‘signal’ that amplifies adaptive responses rather than (or in addition to) serving as a direct fuel. This mechanistic distinction reinforces why acute and chronic supplementation studies should not be interpreted as measuring the same construct and why their results appear divergent despite both being labeled ‘ketone supplementation’.

### 4.3. Applications

The findings of this systematic review offer valuable contributions to professionals in the field of sports science when considering ketone supplementation in endurance sports.

This review summarizes the current trends, highlights areas with promising results, such as cognitive benefits during fatiguing efforts, potential advantages in ultra-endurance events, and adaptive responses during training overload, and outlines the limitations and risks associated with the use of ketones. As such, it may guide practitioners in determining when ketone supplementation may be appropriate, which dosing strategies are supported by evidence, and which variables (e.g., carbohydrate availability and acid–base balance) modulate their effects. From a practical perspective, current evidence suggests that ketone supplementation may be more relevant in specific contexts rather than as a generalized ergogenic strategy. Although some studies report benefits in cognitive performance during fatiguing efforts, ultra-endurance scenarios, or training adaptations, the overall magnitude of these effects appears modest and inconsistent. Acute supplementation with ketone esters may influence metabolic responses; however, performance benefits are not consistently observed and seem highly dependent on exercise intensity, nutritional context, and individual variability. Co-ingestion strategies, particularly with carbohydrates or buffering agents such as sodium bicarbonate, may modulate physiological responses but require further validation to confirm their effectiveness. Athletes and coaches should also consider potential limitations, including gastrointestinal discomfort, inter-individual variability in response, and lack of robust evidence supporting long-term safety and performance enhancement. Grounded in the mechanistic framework discussed above, three practical recommendations emerge: (i) for high-intensity sustained efforts, ketone esters alone are unlikely to enhance (and may impair) performance unless combined with alkaline buffers to mitigate acidosis; (ii) for ultra-endurance or repeated training bouts, the strongest evidence supports chronic protocols that target adaptive signaling (angiogenesis, anti-inflammatory effects) rather than acute metabolic effects; and (iii) for occupational or team-sport contexts involving intermittent or submaximal efforts, acute cognitive and cardiorespiratory benefits may be more readily achievable, though further validation is required. Within these scenarios, individualization based on training status, gastrointestinal tolerance, and specific performance goals remains essential.

### 4.4. Comparison Between Athletes and Non-Athletes

The included studies comprised a broad spectrum of physically active populations. Trained endurance athletes generally show limited performance improvements with acute ketone supplementation, with some studies reporting impairments during high-intensity sustained efforts [32]. In contrast, team-sport athletes engaged in intermittent high-intensity exercise demonstrated more consistent contextual benefits, including improved cognitive function during simulated matches [41] and enhanced recovery between repeated sprint bouts. Occupational groups, such as firefighters, exhibit reduced heart rates during submaximal exercise when wearing personal protective equipment [29], suggesting potential applications in physically demanding occupational contexts. Recreationally active individuals showed metabolic responses similar to trained athletes but with less consistent performance effects, likely due to their lower baseline fitness levels and training status. These differential responses highlight the importance of considering population-specific factors when interpreting the ergogenic potential of ketone supplementation and support the need for tailored recommendations based on training background and performance objectives. The attenuated response observed in highly trained endurance athletes, compared with team-sport athletes or occupational groups, likely reflects pre-existing adaptations in metabolic flexibility. Endurance athletes exhibit chronically elevated mitochondrial density, enhanced fat oxidation capacity, and a more developed capillary network, all of which already optimize oxidative substrate use at baseline. The marginal contribution of an additional oxidizable substrate (βHB) is therefore smaller, in accordance with the ‘law of initial value’ and the principle of diminishing returns. In contrast, less-trained or team-sport athletes may experience a relatively larger benefit because their baseline capacity to oxidize fat and ketones is lower and their reliance on glycolysis during high-intensity efforts is higher. This framework, grounded in established exercise physiology, supports individualized recommendations based on training status and helps explain the population-specific patterns reported in Section 3.5.

### 4.5. Limitations and Future Research

This systematic review has several limitations that affect its internal and external validity. Regarding internal validity, potential bias may have arisen if the inclusion and exclusion criteria were not strictly applied or if the blinding procedures in the original studies were compromised. In addition, in some trials, participants or researchers may have identified the intervention due to its distinctive taste or the occurrence of gastrointestinal symptoms, potentially introducing expectancy effects into the results.

A major limitation is the substantial heterogeneity across the included studies, which precluded a quantitative synthesis through a meta-analysis. Studies differed considerably in supplementation characteristics (ketone salts, ketone esters, and monoesters; acute versus repeated or chronic administration; variations in timing and dosage), exercise protocols (maximal and submaximal tests, time trials of different durations, and sport-specific simulations), participant characteristics, and experimental conditions (e.g., normoxic versus hypoxic environments, nutritional status, and carbohydrate availability). This variability complicates comparisons across studies, may confound the interpretation of findings, and limits both the generalizability and inferential strength of the conclusions. Therefore, the observed effects should be interpreted as context-specific rather than as evidence of a uniform ergogenic response to ketone supplementation in all individuals.

Another important limitation relates to the study population. Although the title and primary research question focused on endurance athletes, the included studies involved a broader range of physically active populations, including team sport athletes, occupational groups with high physical demands (e.g., firefighters), and recreationally active individuals. These populations differ substantially in terms of training status, aerobic fitness, and physiological demands, which may influence their responses to ketone supplementation. Their inclusion was intentional to enhance ecological validity and the applicability of findings to real-world athletic and occupational settings; however, it limited the ability to draw specific conclusions for endurance athletes as traditionally defined. Future systematic reviews applying more restrictive eligibility criteria may provide more targeted insights into this population. Another limitation is the inclusion of both acute and repeated or chronic supplementation studies, which differ in mechanisms and may contribute to heterogeneity in the findings.

External validity is limited by the considerable heterogeneity across studies, including differences in participant characteristics, supplementation protocols, exercise testing modalities, and outcome measures, which restrict the generalizability of the findings to specific populations and conditions. In particular, the inclusion of both maximal and submaximal exercise protocols may have contributed to variability in the outcomes, as these assessments evaluate distinct physiological domains. Furthermore, most studies investigated β-hydroxybutyrate-based supplements, limiting the extrapolation of the findings to other ketone esters or alternative formulations.

Another important limitation is funding transparency and potential conflicts of interest. Many included studies did not clearly report conflict-of-interest statements, hindering a robust assessment of the potential influence of industry sponsorship on the study design, outcome selection, data interpretation, or reporting. Moreover, several studies have been conducted by research groups with potential links to ketone supplement manufacturers. Although such associations do not necessarily indicate bias, their influence cannot be ignored. Publication bias may also be present, with studies reporting positive findings potentially overrepresented relative to those with neutral or negative results. Consequently, the current evidence should be interpreted with caution, and further independent research with transparent reporting is warranted.

Future research should prioritize the standardization of supplementation protocols, direct comparisons between different ketone formulations, and identification of optimal dosing, timing, and co-ingestion strategies. Long-term safety outcomes, including metabolic health, hepatic and renal function, and gastrointestinal tolerance, remain insufficiently investigated in this regard. Further studies should examine the interaction between ketone supplementation and carbohydrate availability, as well as potential contraindications and interactions with other commonly used ergogenic aids.

Finally, controlled investigations in diverse athletic populations—women, elite performers, ultra-endurance athletes, and individuals with varied dietary practices—are needed to determine whether specific subgroups benefit more from ketone supplementation. Understanding these nuances is essential for establishing evidence-based guidelines and maximizing practical applicability. The included studies exhibited substantial heterogeneity in participant characteristics, supplementation protocols, and outcome measures, which limited comparability and precluded quantitative synthesis, such as meta-analysis. Additionally, even though the temporal restriction to studies from 2018 onwards was a deliberate inclusion criterion to ensure methodological consistency, it could limit the comprehensiveness of the evidence synthesis and should be considered when interpreting the findings.

The synthesis provided herein may serve as a foundation for new research, such as meta-analysis studies, as well as experimental projects with larger sample sizes and more homogenous characteristics of the participants, with a sufficient representativity ratio of males and females.

A further methodological consideration is that no formal sensitivity analysis restricted to studies with low risk of bias was conducted. This decision reflects the narrative synthesis design of the present review: without a pooled effect estimate, there is no quantitative result to re-estimate under restricted conditions. However, the impact of risk of bias on the certainty of evidence is addressed indirectly through the GRADE framework (where the ‘study limitations’ domain incorporates risk of bias across the body of evidence) and through the narrative comparison of findings from studies with different risk-of-bias profiles. Inspection of the main findings (Table 4) indicates that the central conclusions (particularly the consistent elevation of β-hydroxybutyrate concentrations and the heterogeneous performance effects) are robust across studies regardless of their methodological quality, supporting the generalizability of the main conclusions within the acknowledged heterogeneity.

## 5. Conclusions

This systematic review of 26 studies shows that exogenous ketone supplementation consistently elevates β-BHB and modifies metabolic variables; however, current evidence does not support consistent ergogenic effects on endurance performance. Findings are highly heterogeneous: most trials report null or negative acute outcomes, while only chronic protocols more consistently show adaptive benefits (e.g., muscle angiogenesis).

For practitioners, evidence supports context-specific rather than generalized use: acute high-intensity efforts require alkaline buffer co-ingestion to be viable; ultra-endurance and training-overload scenarios may benefit from chronic protocols targeting adaptive signaling; and team-sport or occupational contexts may yield preliminary cognitive and cardiorespiratory benefits, although the evidence is limited.

For researchers, two priorities are essential: standardized supplementation protocols specifying dose, timing, formulation, co-ingestion, and exercise modality to reduce heterogeneity and enable cross-study comparability, and population-specific randomized trials stratified by training status, sex, and exercise modality, ideally with pre-registration and a priori subgroup analyses.

In summary, the ergogenic potential of ketone supplementation is highly context-dependent, and progress requires both methodological standardization and more granular and population-specific experimental designs.

## Figures and Tables

**Table 1:** Characteristics of the studies included.

Study	Region	Study Design	Participants	AGE	Female	Intervention	Training Protocol
Acute Supplementation							
Brady and Egan et al. 2024 [21]	Ireland	randomized crossover design	11 males	35.3 ± 7.5	0	KE ± CHO	Treadmill running at five submaximal speeds (10–14 km·h^−1^) for 8 min each, followed by a ramp test to volitional exhaustion
Clark et al. 2021 [22]	North America	Crossover design	9 males	21.9 ± 1.7	0%	βHB salts	30-min steady-state cycling at 60% Wmax → followed by 15-min time trial (max distance).
Dearlove et al. 2021 [23]	UK	randomized, placebo-controlled crossover trial	6 athletes	18–45	NR	Low/high KME	60 min of cycling ergometer exercise consisting of 20-min intervals at 25%, 50%, and 75% maximal power output (WMax). Approximately 40 h before each visit, participants completed glycogen-depleting exercise followed by high-carbohydrate diet (~70% of energy intake).
Dearlove et al. 2021 [24]	UK	controlled, crossover design study	6 athletes	35 ± 5	1 (16.7%)	KE + substrates	1 h cycling at 75% of maximal power on three separate occasions under different substrate availability conditions (KE + CHO, KE + FAT, KE + CHO + FAT)
Evans et al. 2018 [25]	North America	Crossover, open-label (not blinded)	19 cyclists	26.8 ± 7.6	36.84% (7 women)	βHB salts	Incremental cycling test: 8-min stages at 30%, 40%, 50%, 60%, 70%, and 80% of VO_2_max
Evans et al. 2019 [26]	Ireland	Double-blind, Placebo-controlled, Randomized Crossover Trial	8 runners	33.5 ± 7.3	12.5%	KME + CHO	1 h treadmill run at 65% VO_2_max followed by a 10-km treadmill time trial; cognitive tests pre- and post-exercise; 4 visits with 2 main experimental trials
McCarthy et al. 2021 [30]	Canada	randomized, crossover, double-blind, counterbalanced design	19 adults	18–50	47%	KME	30-min cycling at ventilatory threshold followed by a 15-min time trial (3 kJ·kg^−1^ body mass)
McCarthy et al. 2023 [31]	USA	Randomized crossover trial	23 cyclists	31 ± 9	NR	KE	15-min warm-up followed by a 20-min cycling time trial on an ergometer (time feedback only, no performance feedback).
McCarthy et al. 2023 [32]	USA	Randomized, crossover trial	15 athletes	29 ± 12	26.6% (4 women)	KE	Athletes with adequate sleep showed better academic performance and lower stress levels. Sleep quality correlated with overall well-being.
Peacock et al. 2022 [33]	USA	Randomized controlled trial	17 rugby players	20 ± 1	0%	KME	Bath University Rugby Shuttle Test (BURST): 16 × ~5-min blocks including walking, running, rugby-specific drills, and active recovery; high-intensity, sprint, and power tests embedded in each block
Poffé et al. 2020 [34]	Belgium	Randomized parallel-group trial	20 athletes	25 ± 6	0	KE	Simulated cycling race: 3 h intermittent cycling (IMT180′) at intensities relative to lactate threshold, followed by 15-min time trial (TT15′) and maximal sprint to exhaustion
Poffé et al. 2021 [35]	Belgium	Randomized controlled trial	9 cyclists	29 ± 5	0	KE ± BIC	Simulated cycling race: 3 h intermittent submaximal cycling, 15-min simulated time trial, and sprint at 175% lactate threshold
Poffé et al. 2021 [36]	Belgium	Randomized crossover trial	12 cyclists	26 ± 6	0	KE ± BIC	30-min time trial (TT30′) followed by maximal sprint at 175% lactate threshold
Poffé et al. 2021 [37]	Belgium	Randomized controlled trial	14 cyclists	27 ± 6	0	KE ± BIC	four experimental sessions in a normobaric hypoxic facility. Simulated cycling race in hypoxia: 3 h intermittent cycling (IMT180′), 15-min TT, and sprint at 175% lactate threshold; inspired O_2_ reduced from 18.6% to 14.5% during the trial
Quinones and Lemon. 2022 [40]	USA	Randomized controlled trial	13 participants	23 ± 3	52% (12 women)	KS combinations	20-km cycling time trial followed by Wingate test.
Quinones and Lemon. 2022 [41]	USA	Randomized controlled trial	9 participants	30 ± 3	0% (all men)	KME	45-min simulated soccer match (SSM) with three blocks of intermittent high-intensity running and active recovery.
Ramos-Campo et al. 2024 [42]	Spain	Randomized Controlled Trial	28 cyclists	27.46 ± 4.32	0	KE + BIC	Road-cycling stage simulation including: incremental test to exhaustion with gas analysis, warm-up (5 min at 150 W then +30 W·min^−1^ from 180 W), 8-min TT, 30-s sprint, 4.5 h outdoor cycling, second 8-min TT and second 30-s sprint
Waldman et al. 2020 [45]	United States, Ireland	Randomized, Triple-blinded, Crossover Trial	16 males	21.9 ± 1.9	0%	KS	Cycling test: 5 min at 100 W, then +50 W for 3 min in two stages, then +50 W every minute to volitional exhaustion
Waldman et al. 2024 [46]	United States	Randomized, double-blind, 2-condition crossover	12 women	23 ± 3	100%	KME + CHO	Baseline cognitive tests (psychomotor vigilance, task-switching, flanker), followed by 6 × 5-min cycling intervals at 40–65% Wmax, then a 10-km cycling TT; post-exercise repetition of cognitive battery
Repeated or chronic supplementation							
Hiroux et al. 2023 [27]	France	Randomized, controlled trial	32 women	22.2 ± 5	100%	KE 4 weeks	No specific sports tests; focus on caloric restriction period; outcomes: body composition, resting energy expenditure, exercise capacity, appetite hormones, and well-being
Jameson et al. 2022 [28]	USA	Observational study	16	23 ± 3	62.5%(10)	KE repeated 3 days	300 unilateral eccentric knee-extension contractions to induce muscle damage; strength and recovery assessed before and after exercise.
McAllister et al. 2019 [29]	United States	Randomized, Double-blinded, Crossover Trial	9 firefighters	18–39	0%	KS 7 days	day 8: treadmill exercise at 60% VO_2_peak for 35 min in full personal protective equipment
Poffé et al. 2023 [38]	Belgium	Randomized controlled trial	18 cyclists	21.3 ± 2.6	0	KE 3 weeks	3-week endurance overload program with 10 training sessions per week.
Poffé et al. 2023 [39]	Belgium	Double-blind randomized crossover trial	18 cyclists	35.6 ± 7.95	0	KE ultra-endurance	100-km trail run completed, or run to premature exhaustion at ~80 or 60 km
Robberechts et al. 2023 [43]	Belgium	Randomized Controlled Trial	10 cyclists	23 ± 4	0%	KE training/sleep	One 120-min endurance cycling session (8 × 15-min intervals at 60–80% lactate threshold) and one 90-min HIIT session (10-min warm-up at 70% LT, then 10 × 7-min intervals: 3 min at 120% LT + 4 min at 50% LT, finishing with a sprint at 175% LT)
Robberechts et al. 2022 [44]	Belgium	Cross-sectional study	11 cyclists	28.4 ± 5.1	0%	KE.	Simulated 3-h submaximal intermittent cycling followed by a 15-min TT in an environmental chamber (28 °C, 60% RH); fluid intake adjusted to maintain euhydration

KE: Ketone ester, CHO: Carbohydrate, BIC: sodium bicarbonate, KME: ketone monoester, KS: Ketones salts, TT: time trial.

**Table 2:** JBI Critical Appraisal of Randomized Controlled Trials.

Study	Q1	Q2	Q3	Q4	Q5	Q6	Q7	Q8	Q9	Q10	Q11	Q12	Q13
McAllister et al. (2019) [29]	Y	Y	Y	Y	Y	Y	Y	Y	Y	Y	Y	Y	Y
Waldman et al. (2020) [45]	Y	Y	Y	Y	Y	Y	Y	Y	Y	Y	Y	Y	Y
Evans et al. (2019) [26]	Y	Y	Y	Y	Y	Y	Y	Y	Y	Y	Y	Y	Y
Dearlove et al. (2021) [24]	Y	U	Y	Y	N	Y	Y	Y	Y	Y	Y	Y	Y
McCarthy et al. (2021) [30]	Y	Y	Y	Y	Y	Y	Y	Y	Y	Y	Y	Y	Y
Dearlove et al. (2021) [23]	Y	Y	Y	Y	N	Y	Y	Y	Y	Y	Y	Y	Y
McCarthy et al. (2023) [31]	Y	Y	Y	Y	Y	Y	Y	Y	Y	Y	Y	Y	Y
Peacock et al. (2022) [33]	Y	Y	Y	Y	Y	Y	Y	Y	Y	Y	Y	Y	Y
Hiroux et al. (2023) [27]	Y	Y	Y	Y	Y	Y	Y	Y	Y	Y	Y	Y	Y
Jameson et al. (2022) [28]	Y	Y	Y	Y	Y	Y	Y	Y	Y	Y	Y	Y	Y
McCarthy et al. (2023) [32]	Y	Y	Y	Y	Y	Y	Y	Y	Y	Y	Y	Y	Y
Quinones and Lemon (2022) [40]	Y	Y	Y	Y	Y	Y	Y	Y	Y	Y	Y	Y	Y
Quinones and Lemon (2022) [41]	Y	Y	Y	Y	Y	Y	Y	Y	Y	Y	Y	Y	Y
Ramos-Campo et al. (2024) [42]	Y	Y	Y	Y	Y	Y	Y	Y	Y	Y	Y	Y	Y
Robberechts et al. (2023) [43]	Y	Y	Y	Y	Y	Y	Y	Y	Y	Y	Y	Y	Y
Poffé et al. (2023) [38]	Y	Y	Y	Y	Y	Y	Y	Y	Y	Y	Y	Y	Y
Poffé et al. (2020) [34]	Y	Y	Y	Y	Y	Y	Y	Y	Y	Y	Y	Y	Y
Poffé et al. (2021) [35]	Y	Y	Y	Y	Y	Y	Y	Y	Y	Y	Y	Y	Y
Poffé et al. (2021) [36]	Y	Y	Y	Y	Y	Y	Y	Y	Y	Y	Y	Y	Y
Poffé et al. (2021) [37]	Y	Y	Y	Y	Y	Y	Y	Y	Y	Y	Y	Y	Y
Poffé et al. (2023) [39]	Y	Y	Y	Y	Y	Y	Y	Y	Y	Y	Y	Y	Y
Waldman et al. (2024) [46]	Y	Y	Y	Y	Y	Y	Y	Y	Y	Y	Y	Y	Y
Brady & Egan (2024) [21]	Y	Y	Y	Y	Y	Y	Y	Y	Y	Y	Y	Y	Y
Robberechts et al. (2022) [44]	Y	Y	Y	Y	Y	Y	Y	Y	Y	Y	Y	Y	Y

Notes: Y: Yes; N: No; U: Unclear; Q1–Q13: JBI Checklist questions (e.g., Q1: Randomization, Q2: Allocation concealment, Q3: Baseline comparability, Q4: Blinding of participants, etc.).

**Table 3:** JBI Critical Appraisal of Non-Randomized Studies.

Study	Q1	Q2	Q3	Q4	Q5	Q6	Q7	Q8	Q9	^†^ Overall Assessment
Clark et al. 2021 [22]	Y	N	Y	Y	Y	Y	Y	Y	Y	High
Evans et al. 2018 [25]	Y	N	Y	Y	Y	Y	Y	Y	Y	High

Notes: Y: Yes; N: No; U: Unclear; Q1–Q13: JBI Checklist questions. ^†^ Overall Rating: High (7–9 criteria met), Moderate (5–6 criteria), Low (≤4 criteria). Q1: Clear cause and effect (temporal precedence); Q2: Control group present; Q3: Participants similar (confounding); Q4: Similar treatment/care beyond intervention; Q5: Multiple measurements pre and post intervention; Q6: Outcomes measured the same way; Q7: Outcomes measured reliably; Q8: Follow-up complete or differences described; Q9: Appropriate statistical analysis used.

**Table 4:** Main results of the included RCTs and Non-randomized controlled and/or single-blind studies according to time of supplementation.

Study	βHB	Main Findings
Acute Supplementation		
Brady & Egan, 2024 [21]	↑ βHB	= time-to-exhaustion; ↑ running economy (without CHO only) suggesting improved efficiency did not translate into greater maximal exercise tolerance.
Clark et al., 2021 [22]	↑ βHB (dose-dependent)	↓ blood glucose. = power output. ↑ RER, indicating greater reliance on carbohydrates. WBC + KET did not improve performance.
Dearlove et al., 2021 [24]	↑ βHB	moderate positive correlation between pre-exercise intramuscular glycogen and βHB oxidation; higher post-exercise intramuscular βHB abundance in high-fat condition.
Dearlove, Harrison et al., 2021 [23]	↑ βHB	↑ Efficiency at ~2 mM βHB βHB oxidation peaks at 25% Wmax No further βHB oxidation increase above ~2 mM threshold; metabolic flexibility favors cerebral use.
Evans et al., 2018 [25]	↑ βHB (modest)	↓ plasma glucose; ↑ RER and ↑ heart rate at low–moderate intensities. = perceived exertion, muscle efficiency, or lactate. Dose–response relationship exists.
Evans et al., 2019 [26]	↑ βHB	KME did not significantly improve 10-km performance; some individuals exceeded the smallest worthwhile change; no significant differences in running speed or cognitive performance; GI discomfort occurred in both groups (more frequent with KME).
McCarthy et al., 2021 [30]	↑ βHB (high dose)	High-dose ketone monoester acutely ↑ markers of cardiorespiratory stress during submaximal; available data on exercise responses remain limited and equivocal.
McCarthy et al., 2023 [31]	↑ βHB	reduced 20-min TT performance; mechanisms underlying this impairment remain unclear.
McCarthy et al., 2023 [32]	↑ βHB	KE did not increase cardiac output during submaximal exercise despite a modest ↑ heart rate; correcting acidosis with bicarbonate did not change this; VO_2_max and maximal cardiac output similar between conditions, maximal workload was lower with KE
Peacock et al., 2022 [33]	↑ βHB	↑ Sustained high-intensity performance; no significant differences in sprint or power test performance; potential benefit for repeated high-intensity efforts in elite rugby.
Poffé et al., 2020 [34]	↑ βHB (markedly)	Altered glucose, lactate, and free fatty acids; improved high-intensity performance
Poffé et al., 2021 [35]	↑ D-βHB	↓ blood pH and bicarbonate (metabolic acidosis); restored bicarbonate levels by end of IMT180′; ↑ mean power in TT15′; no differences in sprint time-to-exhaustion no GI symptoms.
Poffé et al., 2021 [36]	↑ βHB	neutralized KE-induced metabolic acidosis; no positive effect on high-intensity performance outcomes.
Poffé et al., 2021 [37]	↑ βHB	KE attenuated arterial O_2_ desaturation in hypoxia; no performance improvement. ↑ O_2_ saturation in hypoxia
Quinones and Lemon, 2022 [40]	↑ βHB	↑ 20-km TT (KCT); ↑ peak power (KT) Combined formulation; caffeine contributory but not exclusive factor.
Quinones and Lemon, 2022 [41]	↑ βHB	KME attenuated cognitive decline during high-intensity intermittent exercise.
Ramos-Campo et al., 2024 [42]	↑ βHB	KE + bicarbonate co-ingestion did not improve TT or 30-s sprint performance; altered metabolic/acid–base variables; no significant differences in perceived effort or GI symptoms.
Waldman et al., 2020 [45]	↑ β-OHB	not improvement of cognitive performance during the dual-stress challenge; no ergogenic effect on cognition in this context of high-intensity exercise plus cognitive load.
Waldman et al., 2024 [46]	↑ βHB	↓ glucose and lactate; no differences in 10-km TT time; improved reaction time, processing speed, and accuracy in vigilance and flanker tasks, suggesting a cognitive benefit in trained women;.
Repeated or chronic Supplementation		
Hiroux et al., 2023 [27]	↑ βHB	↓ resting energy expenditure under caloric restriction preserve exercise capacity Helped maintain exercise capacity + REE during caloric restriction; potential for weight management.
Jameson et al., 2022 [28]	↑ βHB	= Strength loss; = cytokines; ↓ TRAIL suggests possible new role in muscle recovery.
McAllister et al., 2019 [29]	↑ βHB (30 min post)	↓ heart rate during exercise, Cardiovascular response modulation without ergogenic benefit in firefighters.
Poffé et al., 2023 [38]	↑ βHB	↑ Muscle capillarization; ↑ pro-angiogenic factors = Endurance (training overload model).
Poffé et al., 2023 [39]	↑ D-βHB (throughout race)	↑ Dopamine; ↓ macrophage infiltration; ↓ AMPK phosphorylation (up to 36 h). = Race performance; ↑ psychocognitive function
Robberechts et al., 2023 (sleep) [43]	↑ βHB (maltodextrin placebo)	= Sleep quality; mostly = blood glucose = Sleep + recovery Many participants misbelieved the placebo contained ketones (potential expectancy bias).
Robberechts et al., 2022 [44]	↑ βHB	↓ Urine production; ↓ NT-proANP Suggests fluid-balance regulation effect of KE; no primary performance benefit.

KE: Ketone ester, CHO: Carbohydrate, βHB: beta-hydroxybutyrate, KME: ketone monoester, KET: Ketone, KT: ketone salts + taurine, RER: Respiratory Exchange Ratio, TT: time Trial, WBC: Whole Body Cooling.

## Data Availability

All data relevant to the study are included in the article or uploaded as online Supplemental Information.

## Supplementary Materials

The following supporting information can be downloaded at: https://www.mdpi.com/article/10.3390/life16071147/s1, Table S1: PRISMA checklist title; Table S2: GRADE assessment.

## Abbreviations

The following abbreviations are used in this manuscript:

AMPK	Adenosine Monophosphate-Activated Protein Kinase
BHB	β-Hydroxybutyrate
CHO	Carbohydrate
EK	Exogenous Ketones
FA	Fatty Acids
KE	Ketone Ester
KS	Ketone Salts
KME	Ketone Monoester
RER	Respiratory Exchange Ratio
VO_2_max	Maximal Oxygen Uptake

## Appendix A

PubMed Search: (Exogenous ketosis OR ketones) and sport Sort by: Most Recent

(((“exogeneous”[All Fields] OR “exogeneously”[All Fields] OR “exogenic”[All Fields] OR “exogenous”[All Fields] OR “exogenously”[All Fields]) AND (“ketosis”[MeSH Terms] OR “ketosis”[All Fields])) OR (“keton”[All Fields] OR “ketones”[Supplementary Concept] OR “ketones”[All Fields] OR “ketone”[All Fields] OR “ketones”[MeSH Terms] OR “ketonic”[All Fields] OR “ketonization”[All Fields] OR “ketonized”[All Fields])) AND (“sport s”[All Fields] OR “sports”[MeSH Terms] OR “sports”[All Fields] OR “sport”[All Fields] OR “sporting”[All Fields])

Translations

Exogenous: “exogeneous”[All Fields] OR “exogeneously”[All Fields] OR “exogenic”[All Fields] OR “exogenous”[All Fields] OR “exogenously”[All Fields]

ketosis: “ketosis”[MeSH Terms] OR “ketosis”[All Fields]

ketones: “keton”[All Fields] OR “ketones”[Supplementary Concept] OR “ketones”[All Fields] OR “ketone”[All Fields] OR “ketones”[MeSH Terms] OR “ketonic”[All Fields] OR “ketonization”[All Fields] OR “ketonized”[All Fields]

sport: “sport’s”[All Fields] OR “sports”[MeSH Terms] OR “sports”[All Fields] OR “sport”[All Fields] OR “sporting”[All Fields].
