# Supplementary material for: Ketone Supplementation in Trained and Physically Active Individuals: Effects on Athletic Performance and Metabolic Variables—A Systematic Review
Source: Life (Basel). 2026 Jul 10;16(7):1147. doi: 10.3390/life16071147 (PMC13412771; doi:10.3390/life16071147)
Supplement: Supplementary file 1 [file life-16-01147-s001.zip › Supplementary Table S2 GRADE.pdf]

Table S2. GRADE assesment

**Outcome**	**Studies (n)**	**Risk of Bias**	**Inconsistency**	**Indirectness**	**Imprecision**	**Publication Bias**	**Certainty**
βHB concentrations	26	Moderate	Not serious	Not serious	Not serious	Not suspected	⊕⊕◯◯ MODERATE
Endurance performance	26	Moderate	Serious	Not serious	Serious	Suspected	⊕◯◯◯ LOW
Cognitive function	6	Moderate	Serious	Not serious	Serious	Suspected	⊕◯◯◯ LOW
Heart rate (cardiovascular response)	5	Moderate	Serious	Not serious	Serious	Cannot be assessed	⊕◯◯◯ LOW
